# Social Media Use and Fear of Missing out: An Exploratory Cross-Sectional Study in Junior High Students from Western Mexico

**DOI:** 10.3390/pediatric16040087

**Published:** 2024-11-18

**Authors:** Manuel Maciel-Saldierna, Ignacio Roberto Méndez-Garavito, Emmanuel Elizondo-Hernandez, Clotilde Fuentes-Orozco, Alejandro González-Ojeda, Sol Ramírez-Ochoa, Enrique Cervantes-Pérez, Berenice Vicente-Hernández, Sergio Jiram Vázquez-Sánchez, Jonathan Matías Chejfec-Ciociano, Gabino Cervantes-Guevara

**Affiliations:** 1 Secundaria Mixta 56 “Juana de Asbaje”, Secretaria de Educación Jalisco, Guadalajara 44200, Jalisco, Mexico; manuel.maciel@jaliscoedu.mx (M.M.-S.); ignacio.mendez@jaliscoedu.mx (I.R.M.-G.); emmanuel.elizondo@jaliscoedu.mx (E.E.-H.); 2 Biomedical Research Unit 02, Hospital de Especialidades, Centro Médico Nacional de Occidente, Guadalajara 44350, Jalisco, Mexico; clotilde.fuentes@gmail.com (C.F.-O.); avygail5@gmail.com (A.G.-O.); vazquezjiram@gmail.com (S.J.V.-S.); jchejfec@live.com (J.M.C.-C.); 3 Department of Internal Medicine, Hospital Civil de Guadalajara Fray Antonio Alcalde, Centro Universitario de Ciencias de la Salud, Universidad de Guadalajara, Guadalajara 44280, Jalisco, Mexico; sramirez@hcg.gob.mx (S.R.-O.); enrique19896@msn.com (E.C.-P.); dra.berenicevicente@gmail.com (B.V.-H.); 4 Departamento de Disciplinas Filosóficas, Metodológicas e Instrumentales, Centro Universitario de Ciencias de la Salud, Universidad de Guadalajara, Guadalajara 44340, Jalisco, Mexico; 5 Departamento de Clínicas, Centro Universitario de Tlajomulco, Universidad de Guadalajara, Tlajomulco de Zúñiga 45641, Jalisco, Mexico; 6 Department of Gastroenterology, Hospital Civil de Guadalajara Fray Antonio Alcalde, Health Sciences University Center, Universidad de Guadalajara, Guadalajara 44280, Jalisco, Mexico; 7 Department of Welfare and Sustaninable Development, Centro Universitario del Norte, Universidad de Guadalajara, Colotlán 46200, Jalisco, Mexico

**Keywords:** adolescence, FoMO, internet, mental health, social media

## Abstract

Background/Objectives: The increased use of social media in Mexico has given rise to the “fear of missing out” (FoMO) phenomenon, especially among adolescents. This study aimed to measure the extent of FoMO among junior high school students in the metropolitan area of Guadalajara, Mexico, during the COVID-19 pandemic. Additionally, this study explored the association between FoMO levels and demographic characteristics, as well as the type and frequency of social media use. Methods: A cross-sectional survey was conducted from November 2021 to January 2022 in four junior high schools. A total of 1264 students (656 females and 608 males) aged 11–16 years completed the Fear of Missing Out Scale, adapted to the Mexican context. Data on demographics, social media usage, and school shifts were collected. Statistical analyses were performed using *t*-tests, ANOVA, and correlation coefficients. Results: The mean FoMO score was 1.79 ± 0.64, with higher scores observed in females (*p* < 0.001) and students attending morning shifts (*p* = 0.001). Significant associations were found between higher FoMO scores and the use of social media platforms like Instagram, TikTok, and Pinterest (*p* < 0.001 for each). The most frequently used social media platforms were WhatsApp (1093), TikTok (828), and Instagram (583). Participants who used social media all week exhibited significantly higher FoMO scores than those who used it only on weekends (*p* < 0.001). Conclusions: FoMO is a significant phenomenon among junior high school students in Guadalajara, Mexico, particularly among females and those who use multiple social media platforms. The findings suggest a need for interventions to manage social media use and mitigate FoMO-related negative health outcomes in this population.

## 1. Introduction

Social media is a term for web-based networks in which users, such as individuals or organizations, belong to online communities, where they can discuss, co-create, or exchange user-generated content, which is posted online, and keep up with current events in the lives of other users [1,2,3]. About 84% of American adults aged 18–30 years use one or more social media platforms. College students are the most digitally driven; about 97% use social media for an average of 3 h per day [1,4]. On average, young Americans check their phones 262 times a day, which is equal to once every 5.5 min [1].

The widespread use of social media reflects the need to appease one’s social needs by communicating with friends and family or by keeping up to date with current events. In February 2022, 45% of respondents to a study conducted in the USA said that they used social networks for news on a regular basis. In that study, social media was the most popular avenue for news consumption among those aged 18–34 years [5].

Social media platforms serve as useful tools; however, the excessive use of these platforms often leads to negative consequences related to emotional distress [6]. One study found that 6.5% of people who use social media excessively have less emotional stability and agreeableness, conscientiousness, perceived control, and self-esteem [7]. Use of these platforms for more than 2 h a day is also known to increase the risk of suicidal tendencies [7,8].

Mexico’s 2020 National Survey on the Availability and Use of Information Technologies in Households (ENDUTIH) found that 65.8% of homes have internet connectivity. Smartphones are the most popular means of accessing the internet and are used by 91.1% of households. Mexicans primarily use the Internet for communication (96.4%), leisure (86.5%), and social networking (81.2%) [9]. In January 2021, there were 100 million social media users in Mexico (Kemp, 2021), which is equivalent to 77.2% of the total population. The average daily time spent on social media is 3 h and 36 min [10].

Before the emergence of social media, people were not exposed to uninvited social activities [11]. Scrolling through a stream of social network postings, images, status updates, and videos displaying the “fun” that one´s peers are having can cause people to experience uneasiness, which has been attributed to a general feeling of “missing out”. The term “fear of missing out” (FoMO) was coined in 2004 and listed in the Oxford Dictionary in 2013 [7]. FoMO is defined as a social media phenomenon characterized by a feeling of anxiety about being absent from a rewarding experience or a fear of missing out on engagement when offline [10]. In a survey from the USA and UK, three-quarters of adults aged 18–34 years reported having experienced FoMO [12].

The worry and discontent produced by FoMO can motivate people to seek connection and engagement, for example, by visiting various social networking websites frequently, which can be considered a reaction to FoMO. Hence, people are directed back to social media, which can lead to the establishment of a vicious cycle. As a result, FoMO can be both a cause and an effect of excessive social media use [1]. According to the self-determination theory (SDT), one of the three innate psychological needs that drive human motivation is the desire for autonomy (the desire to feel connected to others) [12,13]. FoMO can lead to a dependence on external validation and a need for social approval, which can impair a person´s sense of connectedness [12,13,14].

The self-regulatory condition related to FoMO can be triggered by the belief, either momentarily or over time, that an individual´s needs are not being addressed, which can then lead to anxiety, loneliness, depression, sensitivity to social exclusion, and poor academic performance. Individuals whose daily usage of social media exceeds one hour may experience negative effects on their academic performance and potentially develop an addiction to these platforms [15].

FoMO has been associated with harmful effects on health, and experiencing FoMO can have significant mental and physical ramifications, including stress, anxiety, depression, impaired sleep, decreased motivation for academics, excessive mobile phone use, and problematic social media engagement [16]. Additionally, recent research suggests that FoMO can detract from present experiences and lead to reduced satisfaction [16,17]. According to the findings of a Chinese study involving 1288 university students, elevated fear of missing out (FoMO) may impair executive functions, resulting in increased impulsivity and a heightened probability of developing a gambling disorder. These factors may collectively contribute to the progression of addictive behaviors [18].

It has been reported that during the COVID-19 pandemic, specifically in relation to isolation strategies, both social and familial isolation led to higher levels of anxiety and depression, leading the population to increase their use of mobile phones and computers to alleviate psychological stress [19]. FoMO is described as a constant feeling of missing out on other people’s activities, so increased Internet use, social isolation, and reliance on digital means of communication made the COVID-19 pandemic a particular risk situation for increased FoMO [20]. Although it might be thought that, secondary to isolation measures, the absence of travel or social activities could have reduced the feeling of FoMO, this remained focused on virtual meetings or remote events (e.g., concerts) [21]. Liang et al. reported that during the COVID-19 pandemic, psychological stress caused an increase in Internet abuse among young people aged 17–28 compared with what was reported in studies prior to the pandemic, and one of the factors directly associated with the increase in Internet abuse was FoMO [20]. Overall, FoMO was reported to mediate Internet abuse, Internet gaming disorder, and digital media addiction in several studies conducted during the COVID-19 pandemic [16,21].

The working hypothesis of our research was that, in addition to access and time of exposure to social networks, the type of social networks used, and demographic characteristics influence the intensity of FoMO perception. In addition, we assumed that changes in social and family dynamics during the COVID-19 pandemic could increase the time of exposure to the Internet in children and adolescents, which could be useful to facilitate the description of the social media platforms used, as well as to generate a better contrast in the social media platforms that could be most associated with the perception of FoMO. However, due to the negative effects on mental health associated with FoMO, our main objective was to describe the degree of FoMO present in our study population, as well as the use of social networks, which is a phenomenon not described in our population, as well as an age group that has been little studied.

## 2. Materials and Methods

### 2.1. Design

In this cross-sectional, non-experimental study, the recruiting team went to four different junior high schools between November 2021 and January 2022. Purposive sampling, a technique that does not rely on probability, was used for participant recruitment. Students were first briefed on the purpose of this research and the scope of their contribution and then given the opportunity to participate in the survey. Following receipt of their parent´s consent, the students were provided with a link to the online survey. For each classroom, there was a time constraint of 15 min to complete the survey, and instructions were supplied. We collected information on the participants’ gender, age, school year, school shift, number of social media platforms used, number of devices used for social media, family type, number of days and hours per week spent using social media, and number of years as a user of social media.

### 2.2. Sample Size

The sample size was assessed considering an alpha of 0.05 and a beta of 0.20 using Stalcalc in Epi Info TM, version 6.0 (Atlanta: Centers for Disease Control and Prevention), based on the total population of students enrolled in Guadalajara´s public junior high schools. With a student population of 78,134 in the metropolitan region of Guadalajara, a margin of error of 5%, and a degree of confidence of 99%, 661 was determined to be the ideal sample size.

### 2.3. Instruments

The Fear of Missing Out Scale proposed by Przybylski et al. [3] was used as the survey in our study. This instrument is a questionnaire that examines the worry and concerns that an individual may experience about being out of touch with the experiences of their broader social environment. This questionnaire is composed of 10 items designed to measure the dread of missing something/news in a social network and is scored on a 5-point Likert-type scale as follows: 1 = not at all true, 2 = slightly true, 3 = moderately true, 4 = very true, and 5 = extremely true. The items are averaged and assessed directly, with higher scores indicating a stronger presence of the attribute.

The instrument was translated into Spanish with the intent of achieving a linguistic version that is conceptually analogous to Mexico and its culture. Transculturality and conceptualization were prioritized over linguistic or literal equivalency in the translation. The original version of FoMO was translated into Spanish using the reverse translation method, in which two bilingual translators translated the score into Spanish and two other experienced translators independently translated it back into English, resolving the minimal discrepancies by consensus. Subsequently, a specialist in the English language analyzed the questionnaire to identify and correct improper expressions/concepts in the translation. In addition, an expert in Spanish reviewed the surveys in detail. The most appropriate words for the local environment were selected and adapted, given that the respondents were adolescents with a basic education.

### 2.4. Statistical Analysis

Statistical analyses were performed using IBM SPSS Statistics (version 25; IBM Corp., Armonk, NY, USA). Percentages, averages, standard deviations, skewness, and kurtosis were included in the descriptive analyses. For the univariate analysis, a *t*-test was performed, and we performed an analysis of variance (ANOVA) with Tukey’s post hoc multiple-comparisons test. The Pearson and Spearman correlation coefficients were calculated to evaluate the associations between quantitative variables. Statistical significance was set at *p* < 0.05.

### 2.5. Ethical Considerations

Every procedure was performed in a way that ensured that it adhered to the ethical requirements set out by the institution and/or the national research committee, as well as the Helsinki Declaration from 1964, any later updates to it, and other equivalent ethical standards. To respect the participants´ anonymity and maintain their privacy, the surveys were conducted online. In accordance with the General Health Law on Research and the norms of the institution, this study did not present any potential dangers. However, given that the participants were minors, written parental informed consent was obtained prior to the students´ participation. This protocol was approved by both the Ethics Committee of the Secretary of Training and Attention to School Teaching of the State of Jalisco and the Secretary of Education of the State of Jalisco (reference no. 0408/5/2021).

## 3. Results

This study received 1278 responses. After excluding 14 incomplete surveys, a total of 1264 participants (656 girls (51.9%) and 608 boys (48.1%); mean age = 13.38 (standard deviation (SD): 0.98 years); age range = 11–16 years) completed the survey. The participants were categorized as attending morning and evening shifts of school: 954 (74.8%) attended the morning shift, and 319 (25.2%) attended the evening shift. There were 355 (28.1%) students in the first grade, 499 (395%) in the second grade, and 410 (32.4%) in the third grade.

The participants reported using an average of 1.57 ± 0.78 devices to access social media; the most common were smartphones (n = 1203), personal computers (n = 315), and portable computers (n = 172). The average number of social media networks accessed per person was 3.37 (±1.75). The participants connected to social networks for an average of 3.2 (±2.18) days per week. A total of 565 (44.7%) users stated that they used social media only during the weekend, whereas 699 (55.3%) users were online regardless of the day. The demographic characteristics of the sample and social network usage are presented in Table 1 and Table 2.

### FoMO Results and Social Media Behavior

The mean FoMO score for the entire patient sample was 1.79 ± 0.64, with higher scores observed in females (*p* < 0.001) and students attending morning shifts (*p* = 0.001). The scores did not differ significantly between students attending public and private schools (*p* = 0.94). Cronbach’s alpha was used as a measure of internal consistency and obtained a value of 0.76, indicating an acceptable reliability of the questionnaire.

The most frequently used social media platforms were WhatsApp (n = 1093 (86.47%) of the total sample), TikTok (n = 828 (65.51%) of the total sample), Instagram (n = 583 (46.12%) of the total sample), and YouTube (n = 679 (53.72%) of the total sample). Significantly higher FoMO scores were found for participants who used Facebook (*p* = 0.032), Instagram (*p* < 0.001), Messenger (*p* = 0.002), Snapchat (*p* = 0.022), TikTok (*p* < 0.001), Pinterest (*p* < 0.001), and Twitter (*p* < 0.001) compared with those who did not use them. The scores of WhatsApp and YouTube users did not differ significantly from those of nonusers. The scores were significantly higher for participants who used social media during the entire week than for those who limited their use to only the weekends (*p* < 0.001). The complete scores for the Fear of Missing Out Scale according to the demographic group and social network used are presented in Table 3.

The correlations between the scores on the Fear of Missing Out scale and the number of social media platforms used, as measured using Pearson’s r (1270) = 0.25 (*p* < 0.001), were positive but weak, indicating a mild linear relationship between the two variables. The relationship between the number of years using social media and the FoMO scores exhibited a mild linear association, and Pearson’s r (1270) = 0.26 (*p* < 0.001) indicated a modest correlation. The scores did not correlate significantly with the participants’ age (Pearson’s r (1270) = 0.14; *p* = 0.059), the number of devices (Pearson’s r (1270) = 0.15; *p* = 0.057), or the number of days per week using social media (Pearson’s r (1270) = 0.23; *p* = 0.053).

Three ANOVA tests were conducted to assess the interaction effects between the variables of gender, school shift, and frequency of social media use (all week vs. weekends only).

Regarding gender and school shifts and their effects on the FoMO score, the ANOVA was statistically significant (F (3, 1268) = 12.28; *p* < 0.0001). Therefore, a Tukey post hoc test was performed, revealing a statistically significant higher FoMO score in females who attended the morning shift compared with males who attended the morning shift (with a mean of 1.916 vs. 1.756; *p* = 0.0006), females who attended the morning shift compared with males who attended the afternoon shift (with a mean of 1.916 vs. 1.579; *p* < 0.0001), females who attended the afternoon shift compared with males who attended the afternoon shift (with a mean of 1.798 vs. 1.579; *p* = 0.0113), and males who attended the morning shift compared with males who attended the afternoon shift (with a mean of 1.756 vs. 1.579; *p* = 0.0159). A non-statistically significant difference was observed between females who attended the morning shift and females who attended the afternoon shift, as well as between females who attended the afternoon shift and males who attended the evening shift.

Regarding gender and the frequency of social media use (weekdays vs. weekends) and its effect on FoMO score, the ANOVA was statistically significant (F (3, 1268) = 22.16; *p* < 0.0001); therefore, a Tukey post hoc test was performed, observing a statistically significant higher FoMO score in females who used social networks all week vs. females who used them only on weekends (with a mean of 1.979 vs. 1.751; *p* < 0.0001), females who used social networks all week vs. males who used them all week (with a mean of 1.979 vs. 1.827; *p* = 0. 0075), females who used social networks all week vs. males who used them only on the weekend (with a mean of 1.979 vs. 1.593; *p* < 0.0001), females who used social networks only on the weekend vs. males who used them only on the weekend (with a mean of 1.751 vs. 1.593; *p* = 0. 0138), and males who used social networks throughout the week vs. males who used them only on the weekend (with a mean of 1.827 vs. 1.593; *p* < 0.0001). A non-statistically significant difference was reported between females who used social networks only on the weekend vs. males who used social networks throughout the week.

Regarding school shifts and the frequency of social media use and their effects on FoMO score, the ANOVA was statistically significant (F (3, 1268) = 16.48; *p* < 0.0001); therefore, a Tukey post hoc test was performed, observing a statistically significant higher FoMO score in students who attended the morning shift and used social networks all week vs. those who attended the morning shift and used social networks only on weekends (with a mean of 1.92 vs. 1.697; *p* < 0.0001), students who attended the morning shift and used social networks all week vs. those who attended the afternoon shift and used social networks only on weekends (with a mean of 1.916 vs. 1.579; *p* < 0.0001), and students who attended the afternoon shift and used social networks all week vs. those who attended the afternoon shift and used social networks only on weekends (with a mean of 1.853 vs. 1.621; *p* = 0.0113). A non-statistically significant difference was reported between students who attended the morning shift and used social networks all week vs. those who attended the afternoon shift and used social networks all week, students who attended the morning shift and used social networks on the weekend vs. those who attended the afternoon shift and used social networks all week, and students who attended the morning shift and used social networks only on the weekend vs. those who attended the afternoon shift and used social networks only on the weekend.

## 4. Discussion

The evidence is mixed regarding the association between age and FoMO. Most research published in medical journals has focused on college students aged 18–25 years. In one study, there was a significantly higher FoMO scale result among freshmen (first-year university students) and sophomores (second-year university students) than among seniors (fourth-year university students) [3]. In contrast, another study evaluating the FoMO with the same measures as the previous study found more significant sentiments of the FoMO in students in their fourth year of undergraduate education [22]. However, another study reported that age does not have a significant effect on FoMO [23]. In our study, we did not find a significant correlation between age and the results of the FoMO scale.

Evidence relating to the question of whether FoMO is related to gender is diverse and sometimes contradictory. There is evidence in the medical literature suggesting that females are more prone to a higher sense of FoMO [10,24,25]

Nevertheless, some studies that are similar have reported that males experience a higher sense of FoMO [3,22,26]. However, other studies have found no significant associations between gender and FoMO [27,28,29]. In our study, girls had a significantly higher mean score on the FoMO scale than boys. It is important to note that the population of our study was, on average, younger than that reported in other studies.

We found a significantly higher FoMO score for students who use social media during most of the week than those who confine their use to only the weekends. Dibb and Foster found that people use Facebook to overcome feelings of loneliness. However, the use of social media can be a double-edged sword [14]. The time spent on social media is an exercise reading into other people’s lives, which may contribute to FoMO and feelings of a “life not lived”, a life missed out on, or one that they could be leading but are not [14,30]. Song et al. reported that people with greater FoMO use Instagram more than others [30]. We obtained statistically significant results regarding the FoMO score observed among users of certain social media platforms (TikTok, Facebook, Instagram, Pinterest, Twitter, and Messenger), as opposed to WhatsApp, YouTube, and Snapchat users. In the case of WhatsApp and YouTube, we considered that their function is different from the rest of the networks, where WhatsApp is used almost exclusively for direct communication, while on sites like Facebook, Instagram, etc., updates are posted in a general way or to your contacts, about what you do during the day, the places you visit, or other social interactions, so the perception of FoMO, conceptualized as fear of social exclusion, could be more related to this type of networks, where social interactions are disseminated to a group of people simultaneously and in a non-personalized way [7]. Although Snapchat has features more similar to networks such as Instagram or Facebook, in Mexico, it is one of the networks with the least captive audience, so users of this platform could use it mainly to see international trends that do not generate the same latent fear of missing out on “something” related to their close social circle [31]. In the case of YouTube, its main use is for entertainment, and it is not generally used to communicate personal messages or social interactions. Our results are consistent with this observation in that social media networks that allow users to view the lifestyles and activities of others on platforms such as Facebook and Instagram correlated with higher feelings of FoMO [32]. Additionally, having more than one social media account may aggravate the feeling of FoMO, which we found to be significant in our study; however, the correlation was weak, so more studies are needed to confirm or deny this association.

We found no previous research on whether FoMO is associated with the type of school shift. In our study, a higher and significant degree of FoMO was found in students who attended school in the mornings than in the evenings. These differences could indicate that there is a temporal relationship in terms of the sensation of FoMO; however, it must be interpreted carefully, since the scale used in this study is a kind of “snapshot”, which describes a specific moment of psychological state, which could change depending on the moment in which it is taken [3]. In Mexican society, the difficulties or peculiarities of access to public or private education, the differences in this type of education (such as the fact that public schools have two shifts, one in the morning and one in the afternoon, something that is unusual in private primary schools), and the type of social relationship generated by the family structure, accompanied by the great importance of the family in cultural identity, make it relevant to search for possible relationships with new psychosocial phenomena, such as FoMO [7,33,34]. In addition, in Mexico, there is no national regulation regarding the age of access to social networks, which highlights the relevance of studying the specificities of FoMO in our population.

FoMO can disrupt the fundamental psychological requirements outlined by the SDT, which may lead to a vicious cycle of overcommitment, worry, and disconnectedness from others [12,13]. The COVID-19 pandemic exacerbated these problems [18]. In particular, it acted as a mediator for Internet abuse, digital media addiction, Internet gaming disorder, and other activities associated with depression and anxiety that feed the vicious cycle of FoMO [19,20,21].

The results of the ANOVA seem to indicate that although being female, attending the morning school shift, or using social networks throughout the week increases the score on the FoMO scale, both being a woman and using social networks throughout the week seem to be the factors most associated with increasing FoMO regardless of their co-occurrence, while attending the morning school shift has a weaker effect on the perception of FoMO and may be more dependent on other factors.

Research has shown that excessive social media use can exacerbate feelings of FoMO and contribute to negative psychological outcomes, such as anxiety and depression [14,35,36]. Therefore, individuals may benefit from setting boundaries on their social media use, such as restricting usage to specific times of day or limiting the number of platforms they engage with.

This study has some limitations. First, it was conducted on a specific population in an unstudied community, and the FoMO scale results are restricted to that population. Another limitation is that we reported only the total FoMO scores and their associations with the demographic characteristics of the junior high school students; hence, these results need to be compared with caution with other age groups. Future research should examine the possible connections between FoMO and mental and physical harm in a range of settings and populations. One of the main limitations lies in the scale used to measure FoMO. Although there is no consensus on the best scale to measure this phenomenon, and the scale proposed by Przybylski et al. [3] has shown adequate consistency, this scale only shows us a “snapshot” of the individuals at a certain time; hence, it provides little information on the temporal or contextual stability of FoMO (this is because FoMO is taken as an individual difference). In addition, we measured the frequency of social media use only as days of access to social media in a week, considering that the time spent on social media (hours per day) could also be associated with a greater perception of FoMO. We must state that although the translation of the original FoMO scale was carefully performed and adapted to our population, we do not have data on the reliability, dimensionality, and validation of this translation, which is important for the reliability of the use of this tool in this study and subsequent studies.

## 5. Conclusions

In conclusion, our study demonstrates that all participants exhibited varying levels of fear of missing out (FoMO), with significant associations found predominantly among female adolescents, those attending morning school shifts, and users engaging with specific social media platforms, particularly TikTok, Facebook, Instagram, Pinterest, Twitter, and Messenger. Additionally, the frequency of social media use emerged as a critical factor, with higher FoMO levels observed in participants who accessed social networks daily throughout the week compared with those who limited their usage to weekends. These findings highlight the importance of understanding the multifaceted relationship between social media consumption patterns and psychosocial well-being in adolescents. Further research should explore targeted interventions to mitigate the potential mental health impacts of excessive social media use in this vulnerable population.

To lessen the effect of FoMO, it is essential to acknowledge the detrimental effects of this psychological phenomenon and how it can develop. Strategies that make the population aware of the possible negative effects of the conditions derived from the use of social networks, such as FoMO, could enable better detection of these as well as an approach for therapeutic purposes according to personal needs. Given the adverse effect of FoMO on mental and physical health, it is necessary to adopt a multifaceted approach to prevent and manage this phenomenon effectively. Moreover, further research should focus on the incidence and relationships between digital-native generations and the development of evidence-based interventions aimed at fostering healthy relationships between individuals, the internet, and social media use.

## Figures and Tables

**Table 1:** Participants’ demographic information.

	n
Total sample	1278
Incomplete surveys	14
Surveys included	1264
Demographic characteristics	Mean (SD)
Age, years	13.38 (0.98)
Number of networks used	3.37 (1.75)
Number of devices	1.57 (0.78)
Number of days connected per week	3.2 (2.18)
Gender	n (%)
Female	656 (51.9)
Male	608 (48.1)
Type of school	n (%)
Private	64 (5.1)
Public	1200 (94.9)
School shift	n (%)
Morning	954 (74.8)
Evening	319 (25.2)
Family type	n (%)
Extended	307 (24.3)
Nuclear	736 (58.2)
One parent	198 (15.7)
Adoptive	23 (1.8)
Grade	n (%)
First	355 (28.1)
Second	499 (39.5)
Third	410 (32.4)

SD = standard deviation.

**Table 2:** Participants’ social network usage.

WhatsApp	n (%)
Users	1093 (86.5)
Nonusers	171 (13.5)
Facebook	n (%)
Users	388 (30.7)
Nonusers	876 (69.3)
Instagram	n (%)
Users	583 (46.1)
Nonusers	681 (53.9)
Messenger	n (%)
Users	116 (9.2)
Nonusers	1148 (90.8)
YouTube	n (%)
Users	679 (53.7)
Nonusers	585 (46.3)
Snapchat	n (%)
Users	108 (8.5)
Nonusers	1156 (91.5)
TikTok	n (%)
Users	828 (65.5)
Nonusers	436 (34.5)
Devices	
Smartphone	n (%)
Yes	1203 (95.2)
No	61 (4.8)
Portable computer	n (%)
Yes	315 (24.9)
No	949 (75.1)
Computer	n (%)
Yes	172 (13.6)
No	1092 (86.4)
Tablet	n (%)
Yes	142 (11.2)
No	1122 (88.8)
Hours of social media use	n (%)
30 min to 1 h	102 (8.1)
1–2 h	160 (12.7)
2–3 h	205 (16.2)
3–4 h	204 (16.1)
4–5 h	171 (13.5)
5–6 h	129 (10.2)
6–7 h	90 (7.1)
7–8 h	81 (6.4)
9–10 h	50 (4.0)
>10 h	72 (5.7)
Connected only on weekends	n (%)
Yes	565 (44.7)
No	699 (55.3)

SD = standard deviation.

**Table 3:** The FoMO scores according to demographic group and social network platform.

		n	FoMO Mean Score (SD)	Skewness, Kurtosis	Cohen’s d	Hedges’s g	*p*-Value
Gender							
	Male	608	1.71 (0.6)	1.186, 1.633	0.6352	0.6356	<0.001
	Female	656	1.89 (0.7)	1.2, 1.633			
Type of school							
	Public	1200	1.80 (0.6)	1.222, 1.86	0.6363	0.6387	0.904
	Private	64	1.79 (0.6)	1.186, 1.633			
School shift							
	Morning	945	1.84 (0.6)	1.183, 1.633	0.6413	0.6417	0.001
	Evening	319	1.69 (0.6)	1.494, 3.105			
WhatsApp							
	User	1093	1.82 (0.6)	1.292, 2.089	0.6396	0.6400	0.310
	Nonuser	171	1.68 (0.6)	1.129, 1.412			
Facebook							
	User	388	1.86 (0.7)	1.304, 2.256	0.6401	0.6405	0.032
	Nonuser	876	1.77 (0.6)	1.21, 1.637			
Instagram							
	User	583	1.99 (0.7)	1.129, 1.412	0.6160	0.6163	<0.001
	Nonuser	681	1.64 (0.5)	1.292, 2.089			
Messenger							
	User	116	1.98 (0.8)	1.127, 1.478	0.6387	0.6391	0.002
	Nonuser	1148	1.78 (0.6)	1.26, 1.949			
YouTube							
	User	679	1.79 (0.6)	1.129, 1.412	0.6412	0.6416	0.556
	Nonuser	585	1.81 (0.7)	1.26, 1.949			
Snapchat							
	User	108	1.94 (0.7)	1.417, 3.49	0.6399	0.6403	0.022
	Nonuser	1156	1.79 (0.6)	1.213, 1.583			
TikTok							
	User	828	1.86 (0.7)	1.222, 1.86	0.6359	0.6362	<0.001
	Nonuser	436	1.69 (0.6)	1.296, 1.878			
Pinterest							
	User	248	2.03 (0.7)	0.9631, 1.108	0.6313	0.6317	<0.001
	Nonuser	1016	1.74 (0.6)	1.316, 2.167			
Twitter							
	User	103	2.18 (0.8)	1.073, 1.803	0.6315	0.6318	<0.001
	Nonuser	1161	1.77 (0.6)	1.228, 1.67			
Social media use							
	All week	699	1.91 (0.7)	1.087, 1.441			<0.001
	Only weekends	565	1.67 (0.6)	1.544, 3.111			

FoMO = fear of missing out; SD = standard deviation.

## Data Availability

The data presented in this study are available upon request from the corresponding author due to privacy reasons.

## References

[1] Alabri A. (2022). Fear of Missing Out (FOMO): The Effects of the Need to Belong, Perceived Centrality, and Fear of Social Exclusion. Hum. Behav. Emerg. Technol..

[2] Deniz M. (2021). Fear of missing out (FoMO) mediate relations between social self-efficacy and life satisfaction. Psicol. E Crit..

[3] Przybylski A.K., Murayama K., DeHaan C.R., Gladwell V. (2013). Motivational, emotional, and behavioral correlates of fear of missing out. Comput. Hum. Behav..

[4] Gilsenan K. (2021). 2019 in Review: Social Media Is Changing, and It’s Not a Bad Thing. https://blog.gwi.com/trends/2019-in-review-social-media/.

[5] Watson A. (2022). Most Used News Platforms U.S. by Age 2022.

[6] Bui M., Krishen A.S., Anlamlier E., Berezan O. (2021). Fear of missing out in the digital age: The role of social media satisfaction and advertising engagement. Psychol. Mark..

[7] Gupta M., Sharma A. (2021). Fear of missing out: A brief overview of origin, theoretical underpinnings and relationship with mental health. World J. Clin. Cases.

[8] Memon A., Sharma S., Mohite S., Jain S. (2018). The role of online social networking on deliberate self-harm and suicidality in adolescents: A systematized review of literature. Indian J. Psychiatry.

[9] Instituto Nacional de Estadística y Geografía (2023). National Survey on Availability and Use of Information Technologies in Homes (ENDUTIH) 2021.

[10] Kemp S. Digital in Mexico: All the Statistics You Need in 2021—DataReportal—Global Digital Insights. https://datareportal.com/reports/digital-2021-mexico.

[11] Buglass S.L., Binder J.F., Betts L.R., Underwood J.D. (2017). Motivators of online vulnerability: The impact of social network site use and FOMO. Comput. Hum. Behav..

[12] Li L., Griffiths M.D., Niu Z., Mei S. (2020). The trait-state fear of missing out scale: Validity, reliability, and measurement invariance in a Chinese sample of university students. J. Affect. Disord..

[13] Ntoumanis N., Ng J.Y.Y., Prestwich A., Quested E., Hancox J.E., Thøgersen-Ntoumani C., Deci E.L., Ryan R.M., Lonsdale C., Williams G.C. (2021). A meta-analysis of self- determination theory-informed intervention studies in the health domain: Effects on motivation, health behavior, physical, and psychological health. Health Psychol. Rev..

[14] Dibb B., Foster M. (2021). Loneliness and Facebook use: The role of social comparison and rumination. Heliyon.

[15] Sánchez F.J.G., Gutiérrez-García R.A. (2021). Association addictive behavior to social networks high school students: A study in Mexico. Gac. Méd. Caracas.

[16] Hayran C., Anik L. (2021). Well-being and fear of missing out (FOMO) on digital content in the time of COVID-19: A correlational analysis among university students. Int. J. Environ. Res. Public Health.

[17] Akbari M., Seydavi M., Palmieri S., Mansueto G., Caselli G., Spada M.M. (2021). Fear of missing out (FoMO) and internet use: A comprehensive systematic review and meta-analysis. J. Behav. Addict..

[18] Li L., Griffiths M.D., Mei S., Niu Z. (2021). The Mediating Role of Impulsivity and the Moderating Role of Gender Between Fear of Missing Out and Gaming Disorder Among a Sample of Chinese University Students. Cyberpsychology Behav. Soc. Netw..

[19] Marciano L., Ostroumova M., Schulz P.J., Camerini A.-L. (2022). Digital Media Use and Adolescents’ Mental Health During the Covid-19 Pandemic: A Systematic Review and Meta-Analysis. Front. Public Health.

[20] Liang L., Li C., Meng C., Guo X., Lv J., Fei J., Mei S. (2022). Psychological distress and internet addiction following the COVID-19 outbreak: Fear of missing out and boredom proneness as mediators. Arch. Psychiatr. Nurs..

[21] Wang Y., Liu B., Zhang L., Zhang P. (2022). Anxiety, Depression, and Stress Are Associated with Internet Gaming Disorder During COVID-19: Fear of Missing Out as a Mediator. Front. Psychiatry.

[22] Chen B., Liu F., Ding S., Ying X., Wang L., Wen Y. (2017). Gender differences in factors associated with smartphone addiction: A cross-sectional study among medical college students. BMC Psychiatry.

[23] Milyavskaya M., Saffran M., Hope N., Koestner R. (2018). Fear of missing out: Prevalence, dynamics, and consequences of experiencing FOMO. Motiv. Emot..

[24] Balta S., Emirtekin E., Kircaburun K., Griffiths M.D. (2018). Neuroticism, Trait Fear of Missing Out, and Phubbing: The Mediating Role of State Fear of Missing Out and Problematic Instagram Use. Int. J. Ment. Health Addict..

[25] Munawaroh E., Nurmalasari Y., Sofyan A. (2020). Social Network Sites Usage and Fear of Missing Out Among Female Instagram User. Proceedings of the 2nd International Seminar on Guidance and Counseling 2019 (ISGC 2019).

[26] Abel J.P., Buff C.L., Burr S.A. (2016). Social Media and the Fear of Missing Out: Scale Development and Assessment. J. Bus. Econ. Res..

[27] North D., Johnston K.A., Ophoff J. (2014). The Use of Mobile Phones by South African University Students. Issues Informing Sci. Inf. Technol..

[28] Young L., Kolubinski D.C., Frings D. (2020). Attachment style moderates the relationship between social media use and user mental health and wellbeing. Heliyon.

[29] Qutishat M., Sharour L.A. (2019). Relationship between fear of missing out and academic performance among Omani university students: A descriptive correlation study. Oman Med. J..

[30] Song H., Zmyslinski-Seelig A., Kim J., Drent A., Victor A., Omori K., Allen M. (2014). Does Facebook make you lonely?: A meta analysis. Comput. Hum. Behav..

[31] Xataka México Snapchat no Solo no Está Muerto, Sino que su Éxito es tal que Tiene 750 Millones de Usuarios Activos al Mes. https://www.xataka.com.mx/aplicaciones/snapchat-no-solo-no-esta-muerto-sino-que-su-exito-tal-que-tiene-750-millones-usuarios-activos-al-mes.

[32] Pittman M., Reich B. (2016). Social media and loneliness: Why an Instagram picture may be worth more than a thousand Twitter words. Comput. Hum. Behav..

[33] Vázquez-Nava F., Vázquez-Rodríguez E.M., Vázquez-Rodríguez C.F., Betancourt N.V.O. (2019). High School Dropout: Association with Family Structure, Maternal Employment, and Health-risk Habits Among Female Mexican Adolescents. J. Child Fam. Stud..

[34] Binelli C., Rubio-Codina M. (2013). The Returns to Private Education: Evidence from Mexico. Econ. Educ. Rev..

[35] Błachnio A., Przepiorka A., Boruch W., Bałakier E. (2016). Self-presentation styles, privacy, and loneliness as predictors of Facebook use in young people. Pers. Individ. Differ..

[36] Nowland R., Necka E.A., Cacioppo J.T. (2017). Loneliness and social Internet use: Pathways to reconnection in a digital world?. Perspect. Psychol. Sci..

